# The male-focused marital relationship enrichment and sexual well-being interventions: A scoping review

**DOI:** 10.18502/ijrm.v21i12.15035

**Published:** 2024-01-25

**Authors:** Solmaz Abdollahzadeh Sardehaei, Effat Merghati Khoei, Beheshteh Niusha, Zahed Rezaei

**Affiliations:** ^1^Department of Psychology, Faculty of Humanities, Saveh Branch, Islamic Azad University, Saveh, Iran.; ^2^Iranian National Center for Addiction Studies (INCAS), Tehran University of Medical Sciences, Tehran, Iran.; ^3^The Family and Sexual Health Division, Brain and Spinal Cord Injury Research Center (BASIR), Neuroscience Research Center, Tehran University of Medical Sciences (TUMS), Tehran, Iran.; ^4^Asadabad School of Medical Sciences, Asadabad, Iran.

**Keywords:** Sexual health, Men, Marriage, Health promotion.

## Abstract

**Background:**

There is an increasing need for sexual well-being and health promotion strategies that effectively engage men. Researchers have evaluated the efficacy of sexual and reproductive health-related and marital enhancement interventions in male-dominated societies. However, few have focused on culturally appropriate and gender-specific program effects.

**Objective:**

This review aims to evaluate evidence of existing interventions aimed at enriching marital relationships and sexual well-being in adult men.

**Materials and Methods:**

This scoping review was conducted by searching various databases (CINAHL, PsycINFO, EMBASE, Google Scholar, PubMed, Scopus, SID, and Noormags), and other available resources in both English and Persian languages. We located all publications up to January 2023 with no time restriction. Inclusion criteria were studies targeting men in the enrichment of marital and sexual relationships, which focus on marital/sexual enrichment, sexual and reproductive health (SRH) program, passionate relationship, or sexual satisfaction as the main outcomes. PRISMA guidelines were utilized in this review.

**Results:**

Of records 34,405 retrieved by searching, after removing duplicate articles, 8 articles were included based on the inclusion criteria. SRH program was the main focus of 6 articles. Marital and sexual enhancement were common areas of focus in 2 studies. Research methods included 2 semi-experimental, 3 clinical trials, 1 systematic review, 1 content analysis, and a mixed method. According to the assessment result, 4 articles were moderate and 4 were of high quality.

**Conclusion:**

Our findings reveal that a small number of interventions specifically offering SRH or marital and sexual enhancement programs to men should be conducted. As the majority were heterosexual couples, we recommend male-focused programs recognizing men's sexuality.

## 1. Introduction

“Education is not just the learning of facts, but the training of the mind to think and react”. Sexuality education is NO exception (Albert Einstein).

Men have limited access to sexual and reproductive health (SRH) services, or they are not motivated to use the existing programs offered by the communities, workplace, and social networks (1-5). No question that available male-centered programs throughout the settings are various. Culture and the context which a couple lives through, and the meanings attached to sexuality and reproduction make the variety. This verity also explains the couples' range of marital satisfaction (6, 7). The outcomes of good marital quality result in mental health, which means strength (e.g., sexual assertiveness, positive { Adakpa, 2022 #4 } expression), marital satisfaction, physical health, positive self-evaluation, fewer depression and mental illness symptoms, flexibility (e.g., positive sexual attitude, supporting the partner, mutual intimate relations), and self-efficacy (8, 9). The efficacy of SRH-related programs has been investigated in male-dominated societies (10, 11) but not focused on the gender-specificity of the program (12-14). Culturally sensitive gender-focused sexuality education can be more efficient when integrated into marital enhancement training programs advocated by professionals, governments, and SRH services (15-21). However, there is a profound gap in the body of knowledge that needs to be filled with more work. In a systematic review of studies examining interventions to improve SRH in men, 21 studies were identified investigating the effectiveness of sexual education and counseling on SRH-related outcomes (22, 23). In a recent comprehensive review of studies, life satisfaction, positive and negative emotions, and psychological well-being were pointed out as the domains of subjective well-being for men (24). The World Health Organization highlighted the impact of gender norms on men's behaviors so that it emphasizes gender-sensitive programs for men as the targets who need specific training programs to enhance their intimate partnership (25).

Considering the importance of SRH in men, we aimed to conduct this scoping review to get answers to our questions of interest: “what does the literature say about male partners' participation in the SRH programs in order to improve their marital and sexual relationships"?

## 2. Materials and Methods

We conducted this scoping review to identify the span and depth of our topic of study, summarize the evidence, and find gaps in existing literature (11, 16, 26, 27). We did an extensive search using electronic databases and other published resources in English and Persian with no time limitation. We developed a search strategy for MEDLINE, including Medical Subject Headings (MeSH), CINAHL, PsycINFO, EMBASE, Google Scholar, PubMed, Scopus, SID, and Noormags.

### Article selection

We included the publications that evaluated the interventions targeting men. These interventions could be aimed either at improving men's sexual well-being or enhancing their marital relationships. 2 independent reviewers (EMK, SAZ) screened articles for inclusion and exclusion criteria using the customized extraction spreadsheets (Excel, Google Sheets). We employed PRISMA to ease transparent and inclusive reporting of this scoping review (Table I). We used the population, intervention, comparison/control and outcome framework to select interventions that directly relate to the marital and sexual matters of adult men. Our focus is on exploring the effectiveness of marital enhancement and sexuality education programs, and how these programs can improve men's sexual well-being and overall marital satisfaction.

### Inclusion and exclusion criteria 

We selected the studies based on the following criteria: (a) samples: healthy, adult married men; (b) aim of intervention: improving men's marital and sexual relationship and sexual well-being; (c) setting: any countries; (d) outcomes: marital OR sexual commitment, couple's intimacy, passionate relationship, or sexual satisfaction; and (e) study design: according to the goal of this review, all studies regardless of study design the marital relation enhancement and sexual well-being for adult men. We included studies if they introduced any program or intervention to enhance men's marital/sexual relations as well as men's overall sexual well-being. We excluded interventions, offered only to women or couples. We also excluded articles if 1) we could not find a full version of the article to review, and 2) articles in which no English or Persian version was available.

### Data extraction and quality assessment

The characteristics of publication, study design, participants, and study concepts and outcomes, were extracted into Excel. The quality of selected articles was assessed independently by SAZ and EMK using the Joanna Briggs Institute (JBI) (28), a tool for assessing the quality of the selected studies, text, and expert opinion before inclusion in the review. We used JBI to reach a consensus decision on selecting the articles. According to the JBI assessment result, 4 articles were moderate (29-32) and 4 were high quality (33-36). In this review, we did not use meta-analysis due to the heterogeneity of the studies.

## 3. Results

**Figure 1:**
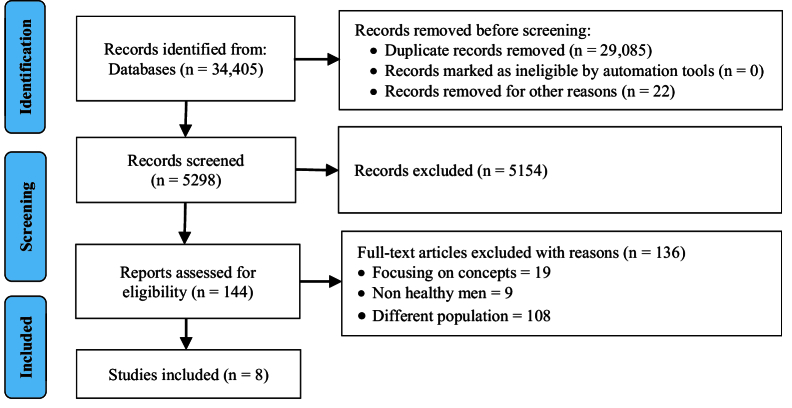
PRISMA flow diagram.

Of 34,405 citations screened, we assessed 5298 articles, and 144 met our inclusion criteria. Consistent with our goal to scope the existing literature to demonstrate the state of interventions in marital and sexual enrichment for men, the study team selected 8 articles for data extraction and summarization (Figure 1).

Findings from the included studies indicated that a variety of strategies were offered within the majority of the articles. SRH program/information, intimate partner violence prevention, human immunodeficiency virus/sexually transmitted infections (HIV/STI)-related topics, gender norms, menopausal health, and prevention programs were the main focus of 6 articles. Marital and sexual enhancements were areas of focus in 2 studies, including marital satisfaction, sexual satisfaction, and marriage enrichment, which show promise for promoting men's sexual health, happiness, and quality of life (Table I). Research methods included 2 semi-experimental studies, 3 clinical trials, 1 systematic review, 1 content analysis, 1 mixed method. Programs included targets from different ethnic/cultural backgrounds. Almost half of the programs were conducted in developed countries 50% and 50% in undeveloped ones. Most often 62.5% of programs focused only on men from American societies and 37.5% from Asian societies.

Among these interventions 37.5% focused on clinical trials. The risk of bias using the Cochrane Collaboration tool: bias due to lack of blinding of study personnel, conflict of interest, and selective reporting of exposures was low. Bias due to exposure misclassification, incomplete exposure data in the selection of participants in the study, and differences in numerator and denominator were medium (Table II).

**Table 1:** Studies included in the scoping review


**Author, Year (Ref)**	**Study design**	**Participant (N#)**	**Intervention, durations, mean-concept**	**Outcome measures**	**Main findings**
**Delkhosh ** ***et al.*** **, 2017 (29)**	A systematic review	Male refugees age > 15	Intervention primary/secondary humanitarian settings IPV prevention. Exploring the effectiveness of existing IPV-related interventions	Primary or secondary IPV	-IPV management policy and associated planning -Decreasing violence against women among refugees, internally displaced persons, and conflict-affected population -Guideline for researchers, policymakers, and strategy developers
**Yoshany ** ***et al.*** **, 2017 (30)**	RCT with a control group follow-up after 2 months	100 Men Age: 45-55 Intervention group #50 Control group#50	Intervention: Education regarding menopausal health 3 sessions (60-min) sessions Conducted using speech	Men's knowledge of menopausal health Women's marital satisfaction Menopause knowledge and ENRICH marital satisfaction questionnaire	The knowledge of menopausal health and women's marital satisfaction scores were ⇧ in the intervention group. Significantly (p < 0.001) 2 months after the intervention
**Bay ** ***et al.*** **, 2013 (31)**	A quasi-experimental Non equivalent control group	80 Married men Age: 20-55	Intervention: Combination of psycho-physiological therapy (Stretching therapy combined + with breathing exercises) 90-120 min 3 days a week 20 sessions conducted in a clinic at the hospital	Sexual satisfaction The ENRICH questionnaire	-The intervention group post-test scores ↑ -Follow-up test scores were ↑ for the intervention group -But no significant statistical difference was observed
**Pulerwitz ** ***et al.*** **, 2006 (32)**	A quasi-experimental 6 month follow up	780 Men Age: 14-25	Intervention: Horizons and Institute Promundo Intervention 1: Interactive group education sessions Intervention 2: Community-wide “lifestyle” social marketing campaign to promote the usage of condom Intervention 3: Morro dos Macacos, a delayed intervention that followed the control period 6 group education sessions 2 supervisors held weekly Conducted by the group education sessions	-Key HIV/STI-related topics -Gender norms attitudes -The gender-equitable men scale	-A variety of key HIV/STI-related outcomes improved -Reduced HIV/STI risk was associated with ⇧ agreement with more equitable gender norms -Couple communication about HIV/AIDS remained approximately high -Reported STI symptoms ↓
**Miller ** ***et al.*** **, 2020 (33)**	Cluster RCT With a control group Follow up: 3-month 9-month	866 Boys Age: 13-19	Intervention Manhood 2.0 a gender-transformative program and job-readiness training program for the control group 6 sessions of 3 hr Once or twice a week Conducted by youth-serving organizations and community	Primary outcome: ARA or SV perpetration exploratory outcome: sexual/physical intimate violence for a partner, non-partner SV, any SV, sexual harassment, dating or cyber-sexual abuse, and incapacitated sex. Secondary outcomes: attitudes about gender equality, ARA recognition, intention to intervene with peers, condom negotiation self-efficacy. A scale to measure participants' views on gender norms/A scale to measure the ability of participants to understand harmful actions committed against a partner as abusive/A scale to measure the probability that a participant would intervene when witnessing damaging behaviors in male peers. A scale for assessing participants' confidence in negotiating condom use with a partner. A scale for assessing participants' views on the use of condoms and contraceptives	-Gender-based violence was ↓ -The difference in reduction was not significant. -There was a primary outcome change in SV participant-level perpetration or adolescent. -Relationship abuse at T3 (ARA)
**Manlove ** ***et al.*** **, 2022 (34)**	A mixed-methods study with a control group Follow up: Only a post-intervention follow-up group	110 Men Age: 15-18 IG#56 CG#54	Intervention: 2.0-an SRH program (group-based+ after-school) targeting young Black and Latino men. (Evaluating the feasibility, quality, and preliminary efficacy) 6 sessions monthly Conducted by: youth center researchers/transcribed recordings (450 min)	Self-efficacy contraception knowledge; SRH information; positive attitudes for supporting partners in the prevention of pregnancy. Gender norms (GEM scale) social competence scale	-The manhood 2.0 program is feasible for delivering unintended pregnancy prevention programming to young men. -SRH information; contraception knowledge; positive attitudes for supporting partners in pregnancy prevention; self-efficacy in partner communication about sex; discussing program content with friends and family; and social competence and support ↑
**Miller ** ***et al.*** **, 2012 (35)**	Cluster RCT with a control group Follow up 3 months	2600 Boys Age: < 18	Intervention: Exploring the effectiveness of a prevention program (dating violence perpetration) among young male athletes and coaches. 60-min training for coaches 11 “Training Cards”. (10-15 min) weekly Conducted by a trained violence prevention advocate to introduce the coaches Kit	Primary outcomes: gender-equitable attitudes and intentions to intervene, abusive behaviors recognition. Secondary outcomes: abuse perpetration and explored bystander behaviors, values for intentions to intervene, gender-equitable attitudes, and scales of negative bystander intervention	-Improved intentions to intervene, abusive behaviors recognition, and positive bystander intervention. DV perpetration and abuse perpetration were reduced. -No notable changes in gender-equitable attitudes + identification of abusive behaviors + DV perpetration were observed
**Hurt ** ***et al.*** **, 2012 (36)**	A mixed method study 2 focus groups NO CG	12 Men Age: 26-50	Intervention: The ProSAAM a 5-yr study 3 programs + 2 wk of skills practice (between each session) conducted by 2 African American male facilitators At a church	Participants' enthusiasm for taking part in the marriage-strengthening program (ProSAAM)	Hearing the voices of a sample of the recruited population and searching for feedback about participating in a similar marriage enrichment program helped promote potential participants' enthusiasm for this marriage-strengthening program
IPV: Intimate partner violence, RCT: Randomized control trial, HIV: Human immunodeficiency virus, STI: Sexually transmitted infections, AIDS: Acquired immunodeficiency syndrome, ARA: Adolescent relationship abuse, SV: Sexual violence, IG: Intervention group, CG: Control group, SRH: Sexual and reproductive health, Gem: Gender-equitable men, DV: Dating violence, ProSAAM: Program for strong African American marriages					

**Table 2:** The risk of bias using the Cochrane Collaboration tool


**1. Bias in the selection of participants into the study**	Medium
**2. Bias due to lack of blinding of study personnel**	Low
**3. Bias due to exposure misclassification**	Medium
**4. Bias due to incomplete exposure data**	Medium
**5. Bias due to selective reporting of exposures**	Low
**6. Bias due to conflict of interest**	Low
**7. Bias due to differences in numerator and denominator**	Medium
**8. Other bias**	Non

## 4. Discussion

In this scoping review, we visually presented the result of complex and limited studies in the field of male-focused marital and sexual well-being interventions. As reflected by the small fraction that was accessible during our review, our findings suggest a prevalent lack of transparency in the conduct and reporting of male-focused programs in enhancing men's marital and sexual relationships, in developing countries. Several explanations have been pointed out for this limitation, such as gender norms that are dismissed in many research contexts. Some reports support our findings. These findings have pointed out gender norm-related beliefs and attitudes as influencing factors on men's participation in research (37). For instance, many family planning programs focus on women's participation, while most contraceptive methods are directly utilized by men (e.g., male condoms, vasectomy, withdrawal) (38).

Others argue that the lack of men's participation is justified because women are considered contraceptive users or must learn more than men because they are the ones falling pregnant. It seems that lack of attention to gender norms in the programs related to SRH or marital satisfaction enrichment makes the intervention ineffective and demotivates men from active participation in the related research.

Most programs, in male-dominated societies in particular, are implemented based on the perspective that women must learn to serve their male partners (38, 39), women should learn how to gratify their husbands sexually (40), or women should learn to elevate the quality of their marital life because women are considered vulnerable than men (41, 42). Other scholars highlighted the gender differences in marital construction (42). In this line, some emphasize gender norms and their powerful influence throughout marital life. In this study with couples, women's roles are significant in marital problem-solving, and somehow men are drawn out of the scene (43). In conservative and androcentric cultures, some connotations affect the societal mindsets and encourage the belief that the man is the breadwinner, and the woman is the counterpart who should learn to perform her duties as the housewife, mother, etc. (44). This paradigm can increase men's feelings of isolation resulting in limited participation of men in research (45). Findings identified that sociocultural and psychological norms are also influencing matters. The Nepali men are not active in implementing SRH programs (46).

The findings of this qualitative study highlighted the absence of education, myths, and domination of women as healthcare providers in most clinical settings as the determinants of the lack of men's involvement in SRH programs. Others explained the lack of men's involvement in SRH programs due to the impact of political, economic, and organizational factors. In a study conducted in Uganda, where men's involvement in SRH implementation is still low, the researcher found some barriers in applying policies driving men's participation in SRH programs such as `gaps between practice and policy', `skills and resources', `inadequate key actor participation', and `types of dissemination' (47). The health system, in terms of health providers' unfriendly service delivery and socioeconomic and cultural issues, are also highlighted as the influencing factors in the level of men's participation in mother-to-child transmission of HIV (48), as an important part of SRH programs. In line with others, we also emphasized on having more studies and research-based investments in men as agents of change (49) or high impact on SRH promotion.

## 5. Conclusion

The profound novelty effect of this review is a combination of the existing data with new knowledge, which is needed to fill the knowledge gap, particularly in male-dominant societies. Ideas from different fields of research in SRH and marital satisfaction enrichment targeting men can lead to completely novel discoveries with potential applications of the relevant program implementation. Translational applications of sexology to psychology will raise enormous advances in the diagnosis and management of numerous problems among couples.

Most reports originate from developed societies, and fewer studies are conducted on men in developing countries. There are some explanations for the limited male-focused research-based pieces of evidence. Our findings reveal the necessity of gender-specific studies in the future and implementing interventions and programs focusing on men's participation in conservative and male-dominated societies.

##  Conflict of Interest

The authors do not mention any conflict of interest in this article.
